# Modulation of Gut Microbiome as a Therapeutic Modality for Auditory Disorders

**DOI:** 10.3390/audiolres13050066

**Published:** 2023-10-10

**Authors:** Dimitri A. Godur, Alexa J. Denton, Nicolas Eshraghi, Jeenu Mittal, Jaimee Cooper, Moeed Moosa, Rahul Mittal

**Affiliations:** 1 Department of Otolaryngology, Hearing Research and Cochlear Implant Laboratory, University of Miami Miller School of Medicine, Miami, FL 33136, USA; dgodur@med.miami.edu (D.A.G.); adent003@med.fiu.edu (A.J.D.); nxe211@miami.edu (N.E.); j.mittal@med.miami.edu (J.M.); jcooper12@student.touro.edu (J.C.); mmm428@miami.edu (M.M.); 2 Herbert Wertheim College of Medicine, Florida International University, Miami, FL 33199, USA; 3 School of Medicine, New York Medical College, Valhalla, NY 10595, USA

**Keywords:** microbiome, sensorineural hearing loss, otitis media, tinnitus, probiotics

## Abstract

The gut microbiome has been shown to play a pivotal role in health and disease. Recently, there has been increased interest within the auditory community to explore the role of the gut microbiome in the auditory system and its implications for hearing disorders such as sensorineural hearing loss (SNHL), otitis media, and tinnitus. Studies have suggested that modulating the gut microbiome using probiotics as well as with diets high in monounsaturated and omega-3 fatty acids is associated with a reduction in inflammation prevalence in auditory disorders. This review aims to evaluate the current literature on modulation of the gut microbiome and its effects on otological conditions. The probiotic conversion of nondigestible carbohydrates into short-chain fatty acids has been shown to provide benefits for improving hearing by maintaining an adequate vascular supply. For acute and secretory otitis media, studies have shown that a combination therapy of probiotics with a decreased dose of antibiotics yields better clinical outcomes than aggressive antibiotic treatment alone. Gut microbiome modulation also alters neurotransmitter levels and reduces neuroinflammation, which may provide benefits for tinnitus by preventing increased neuronal activity. Further studies are warranted to evaluate the efficacy of probiotics, natural health products, and micronutrients on auditory disorders, paving the way to develop novel interventions.

## 1. Introduction

The gut microbiome refers to the collection of fungi, viruses, and bacteria residing inside the gastrointestinal (GI) tract [1,2,3,4]. These organisms provide great benefits in maintaining the health and normal physiological function of the human body. The gut microbiota play a crucial role in health and disease [5,6,7,8,9,10,11,12,13,14,15] and can be modulated through our diet to confer health benefits. Much like the ways in which the gut microbiota can impact the nervous system through the gut–brain axis [16], a previous review shed light on the connections between the gut and inner ear through the blood–labyrinth barrier, a semipermeable partition between the intricate blood vessel capillaries and the endolymph and perilymph of the inner ear [17]. Thus, the blood–labyrinth barrier can regulate the flow of circulating cells, oxygen, fluids, metabolites, and ions from the gut into the inner ear [17]. 

Studies have shown that chronic inflammation is at the root of various auditory disorders, including sensorineural hearing loss (SNHL), otitis media, and tinnitus [16,18]. Recent studies have demonstrated how maintaining a healthy gut microbiome via an increased intake of monounsaturated as well as omega-3 polyunsaturated fatty acids and a decreased intake of saturated fat can help to suppress host inflammatory pathways, including those present in the auditory system [19,20]. Furthermore, these diets facilitate the maintenance of homeostasis by providing protection against oxidative stress, inflammation, platelet aggregation, and modification of hormones and growth factors contributing to disease pathogenesis [16]. Capitalizing on the link between gut dysbiosis and auditory disorders, low-risk and accessible treatments can be implemented to manage these diseases. The use of probiotics, administered orally or intranasally, has been shown to alter neurotransmitter levels and may be helpful for the treatment of auditory disorders. The objective of this review article is to discuss the recent advancements in developing novel therapeutic modalities for auditory disorders such as SNHL, otitis media, and tinnitus based on the modulation of the gut–inner ear axis by targeting the gut microbiome (Table 1). 

## 2. Probiotic and Prebiotic Use in Medicine

According to the World Health Organization (WHO), probiotics refer to live microorganisms, such as bacteria and yeast, that lead to a health benefit when consumed in an adequate quantity [39,40]. In general, their beneficial effects augment and maintainhealthy microbiota [41,42]. Various studies have evaluated the use of probiotics, with the most dominant microbial strains used being *Lactobacillus* genera, *Bifidobacterium* genera, *Lactococcus* spp., *Streptococcus thermophilus*, and the yeast *Saccharomyces boulardii* [43]. The mechanism of action of each of these species and their effects on gut health vary; however, it has been suggested that the various fermentation properties and resultant metabolites enhance the inherent immune system [44]. As a result, they are thought to reduce the colonization of pathogenic bacteria by enhancing the phagocytic activity of leukocytes and stimulating antibody production [45]. 

### 2.1. Prebiotics

Prebiotics are micronutrients that foster the growth and activity of a beneficial gut microbiome. They are shown to be useful in diseases affecting the GI system, immune system, and nervous system in humans [46,47,48,49,50,51]. Harmful microbes which stimulate the immune system can be decreased and the cytokine environment can be modulated with prebiotics. Fructo-oligosaccharide (FOS) administration has been shown to modulate the immune response to vaccines in children. In addition, neural pathways and endocrine pathways have been shown to be modulated with prebiotics. FOS and galacto-oligosaccharides (GOS) have regulatory effects on neurotransmitters and synaptic proteins [46]. The beneficial effects of prebiotics may be used for the treatment of auditory disorders. While there are no approved clinical applications for prebiotics in otological conditions, there are preclinical animal model investigations that have shown positive results in reducing oxidative stress and consequently improving hearing function [52]. 

### 2.2. Clinical Uses of Probiotics and Prebiotics

Probiotics and prebiotics can be used for the prevention and even the treatment of specific diseases, as they can regulate systemic and local immune system activity [16]. Some probiotics even propagate wound healing and minimize the risk of perioperative infection, which has become an increasingly relevant complication of head and neck procedures [19]. Probiotics and prebiotics have been used as a therapeutic tool for various GI disorders due to their anti-inflammatory and antimicrobial effects. More recently, studies have shown the efficacy of probiotics in otolaryngological conditions such as rhinosinusitis, obstructive sleep apnea, and head and neck cancer; however, there is still a paucity of the literature evaluating their utility in otological disorders [19,53,54].

## 3. Gut Health and Sensorineural Hearing Loss (SNHL)

Studies have observed that the “leaky gut” has also led to cases of SNHL, particularly when resulting from a high-fat diet, which induces gut dysbiosis and increases the permeability of the blood–labyrinth barrier [55]. Gut dysbiosis is an imbalance in the microbiota in the gut and serves as a predisposing factor for several disorders [17]. Bacterial metabolites can enter the bloodstream from the gut and cross the blood–labyrinth barrier, causing chronic inflammation of the cochlea from the release of pro-inflammatory cytokines and the production of reactive oxygen species [17,55]. Cases of SNHL were especially prevalent as a secondary condition for patients with inflammatory bowel disease [17]. Sujlana and colleagues found that in a cohort of children with profound hearing loss, the administration of probiotic mouthwash decreased the risk of periodontal infection, although further studies are warranted to identify changes in hearing outcomes [56]. 

### 3.1. Fatty Acids and SNHL

The probiotic conversion of nondigestible carbohydrates into short-chain fatty acids could reduce hearing loss. A study by Gopinath and colleagues demonstrated that polyunsaturated fatty acids help to maintain an adequate vascular supply to the cochlea and thus promote healthy auditory function [21,22]. These polyunsaturated fatty acids also attenuate inflammatory processes, decrease blood pressure, and improve vascular reactivity and endothelial function. Curhan and colleagues also demonstrated that the consumption of two or more servings per week of fish as well as a greater intake of long-chain omega-3 fatty acids are associated with a lower risk of hearing loss in women [23]. Further studies are warranted to observe the effects of omega-3 fatty acid intake on aging populations to observe potentially improved hearing outcomes, as elderly populations are the most susceptible to hearing loss [22].

### 3.2. Inflammatory Bowel Disease and SNHL

Addressing the underlying inflammatory bowel disease when applicable can be a first-line defense in treating auditory disorders through methods such as antibiotics or fecal diversion [55]. While some antibiotics have been known to be a risk factor for inflammatory bowel disease, a combination therapy implementing decreased doses of antibiotics alongside probiotic administration can be further explored, based on their positive results in children as well as decreased antibiotic prescriptions following the administration of probiotics. Furthermore, a healthy diet is essential to help preserve hearing by modulating the health of the gut microbiome. Unfortunately, such a diet greatly conflicts with modern nutritional habits that commonly lead to obesity, poor metabolism, and inflammation [26]. Notably, omega-3 fatty acids, polyphenols, and micronutrients have demonstrated some of the greatest health benefits in modulating gut microbiota [57,58]. Currently, the Mediterranean diet has been shown to modulate the gut microbiota by increasing its diversity and stability, along with maintaining the activity of host immune functions [24,25]. Therefore, adopting the Mediterranean diet may help to prevent gut dysbiosis and inflammatory bowel disease, which can improve GI health and potentially improve hearing outcomes [26]. 

## 4. Acute Otitis Media

Viral, bacterial, and fungal infections as well as allergies are the most common causes of acute otitis media (AOM), which is one of the most prevalent health concerns in children [59]. AOM is diagnosed in approximately 80% of children and is commonly associated with dysfunction of the eustachian tube [16]. Antibiotic treatment has been the cornerstone of therapy for AOM, but it has also been associated with adverse effects (AEs) including vomiting, diarrhea, and rash as well as neurological complications later in life such as a predisposition to autism spectrum disorder [60,61]. Studies have continued to suggest that the incorporation of probiotics in treatment can augment antibiotic use by reducing AEs, improving antibiotic function, and enhancing mucosal immunity [62].

### 4.1. Probiotics and AOM

A study by Scott and colleagues demonstrated that probiotics may be an adequate preventative therapy for reducing the incidence of AOM. Investigators used 17 randomized control trials (RCTs) of children with AOM taking probiotics in comparison to control groups [27]. Eleven groups used *Lactobacillus*-containing probiotics and six groups used *Streptococcus*-containing probiotics. The study concluded that in children who were not prone to acquiring AOM, the incidence of developing AOM decreased by two-thirds when taking these probiotics compared to those not taking the probiotics [27]. Furthermore, the probiotics also aided in decreasing the number of children taking antibiotics for other infections. However, the probiotics did not help children who were already prone to acquiring AOM [27]. While the study did not provide a concrete definition for children “prone” to AOM, it suggested that the differences were likely due to clinical, pathological, and immunological factors [27]. Further investigations of these factors should be conducted particularly in immunocompromised children, comparing their outcomes of AOM after taking probiotics versus not, as well as with the results in healthy children. Different combination doses of probiotic and antibiotic therapy should also be considered for further study. 

Another treatment being studied is the use of *Streptococcus salivarius* K12, a heavily studied oral commensal streptococcus that has been found to inhibit the growth of pathogens in the oral cavity and nasopharynx while maintaining a high safety profile [28]. Di Pierro and colleagues administered oral K12 tablets daily to 22 children prone to AOM for 90 days. After being examined for episodes of AOM, the children were also subjected to tone audiometry, tympanometry, endonasal endoscopy, otoscopy, and tonsillar examinations [28]. Not only were the episodes of AOM reduced, but positive results were also seen across the other clinical outcomes. The researchers thus concluded that K12 may play an important role in reducing the occurrence of AOM [28], a promising result that should be further investigated with a greater sample size. 

A study of toddlers at daycare centers conducted by Stecksen-Blicks and colleagues investigated the use of probiotics and fluoride-supplemented milk. In this investigation, 248 children aged 1–5 were randomly assigned to one of two groups. The intervention group received milk supplemented with *Lactobacillus rhamnosus* and fluoride, while the control group received standard milk [29]. While the number of absent sick days did not differ between the two groups, in those who received the full 21-month course, the use of probiotics significantly reduced the otitis media infection duration by 60% based on a decrease in the antibiotic therapy duration [29]. Similarly, the Stecksen-Blicks study demonstrated that milk supplemented with both probiotics and fluoride consumed once daily helped to prevent OM [19]. 

Rautava and colleagues investigated the daily supplementation of a formula with *Lactobacillus rhamnosis* GG and *Bifidobacterium lactis* Bb-12 probiotics for infants between 2 and 12 months [30]. The largest reduction in the number of otitis media episodes was found within the first seven months for children who consumed the probiotic-supplemented formula. Only 22% of infants receiving probiotics experienced AOM, while 50% of those receiving the placebo experienced AOM [30]. In addition, the rate of antibiotic usage was reduced from 60% in placebo subjects to 31% in probiotic subjects, further suggesting the benefits of probiotic consumption in reducing the risk of early AOM [30]. These results suggest that aggressive antibiotic intervention may not necessarily be the most efficacious treatment, further hinting at the benefits of a probiotic/antibiotic combination therapy. 

### 4.2. Nasal Administration of Probiotics

A balanced flora in the nasopharynx helps to prevent colonization by pathogenic strains and the subsequent otitis media that occurs. In addition, nasal spray remedies have been found to produce clinical benefits in both alpha-*streptococci* strains as well as *Streptococcus salivarius* strains [16]. A study by Roos and colleagues found preventive effects on AOM in which 42% of subjects did not show recurrence when administered one of five strains of *Streptococcus* probiotic via a nasal spray, in comparison to only 22% that did not show recurrence in the placebo group [31]. In another study, investigators concluded that in the sub-group of children who were colonized with probiotic flora in the nasopharynx, the colonized children experienced fewer episodes of AOM and subsequently required less antibiotic administration when compared to those without colonization [32]. So far, no studies have yet directly compared the effects of probiotics administered orally versus trans-nasally in reducing AOM. However, the results tend to indicate better outcomes when probiotics are administered trans-nasally [47]. Thus, studies investigating the outcomes with different methods of probiotic administration should be further considered.

## 5. Secretory Otitis Media

Secretory otitis media (SOM) is another common disease in children that is also associated with effusion in the middle ear cavity and is typically a sequela of AOM. Both orally and nasally administered *Streptococcus* strains yielded promising results in treating SOM. Oral administration of *Streptococcus salivarius* K12 also conferred improvements in tone audiometry and the palatine tonsil size [19]. K12 is known to produce two lantibiotics that inhibit the growth of *Streptococcus pyogenes*, *Streptococcus pneumoniae*, and *Moraxella catarrhalis*, all of which are involved in the pathogenesis of AOM and pharyngotonsillitis [28]. Another study demonstrated increased spontaneous recovery in children with SOM when taking *Streptococcus sanguinis* [16]. In a double-blind, randomized, placebo-controlled study, investigators found significant protection against SOM in children who took the probiotic versus those who took the placebo [16]. Multiple studies also noted decreases in SOM following administration of a probiotic nasal spray containing *Streptococcus sanguinis*, which modulates the nasal microbiota instead of the gut, and is subsequently a promising area of study [16].

## 6. Natural Health Products in Treating Otitis Media

Studies have also investigated the use of natural health products for reducing the number of episodes of AOM. Some of these products, which are generally regarded as safe, include echinacea and xylitol. Echinacea, a North American coneflower, is one of the most popular herbal remedies used for the treatment of upper respiratory infections such as the common cold. It is thought that it enhances the immune system and has anti-inflammatory properties; however, the current literature suggests there may only be a minimal benefit in reducing symptom length and severity [63]. Some studies have further evaluated echinacea’s use as a treatment for AOM. One study by Cohen and colleagues found that echinacea combined with propolis and vitamin C significantly reduced the number of AOM episodes by 68% in comparison to the placebo. However, when studying echinacea alone, there was no decreased risk of AOM in children [33,34]. More recent randomized control trials and meta-analyses found that children with upper respiratory infections who took echinacea did in fact have a significantly decreased risk of complications including AOM [45,64,65]. This suggests that there may be a benefit in using echinacea to prevent AOM. 

### Xylitol and AOM

Xylitol, a natural sugar used in chewing gum and found in fruits, has also been theorized to treat AOM. Evidence has suggested that in vitro use of xylitol is able to inhibit the growth and attachment of *Streptococcus pneumoniae* and *Haemophilus influenzae,* common etiologies of AOM, to nasopharyngeal cells [47]. One of the earlier studies, conducted in 1996, demonstrated that compared to controls, there was a 40% reduction in otitis media with chewing gum, a 30% reduction with syrup, and a 20% reduction with a lozenge, all with fewer antibiotics needed for further treatment. However, there were some side effects such as diarrhea and abdominal discomfort [35]. In contrast to the results from researchers studying echinacea, Azarpazhooh and colleagues concluded that xylitol could help reduce the risk of AOM in children with no active upper respiratory tract infection [63]. 

Although both echinacea and xylitol have produced varying results, it has been suggested that echinacea can be used to prevent AOM in patients who already have active upper respiratory infections and xylitol may be used in patients with isolated AOM infections. Further studies with large cohorts should be conducted to confirm the efficacy and uses of these therapies in regard to AOM.

## 7. Tinnitus

Tinnitus is an auditory disorder that affects approximately 15–20% of people in their lifetime, especially those with underlying hearing loss, and manifests via an etiology that is not fully understood. Some evidence suggests that tinnitus can be triggered by an increase in auditory neuronal activity. A study reported a significant reduction in gamma-aminobutyric acid (GABA), a major inhibitory neurotransmitter, in the auditory cortex of tinnitus patients, which is associated with an increase in neuronal activity [66]. Serotonin levels were also found to be increased in cases of tinnitus, whereas dopamine levels were decreased [66]. Other evidence has suggested that neuroinflammation remains a major underlying cause of tinnitus, with animal models demonstrating an increase in cytokine TNF-alpha as a marker of inflammation and its subsequent inhibition or genetic mutation alleviating the behavioral phenotypes associated with tinnitus [66]. Current treatments for tinnitus include systemic and intratympanic (IT) steroids, hearing aids, and hearing therapies as well as various antidepressants, anticonvulsants, anesthetics, and surgical interventions. Steroids may cause systemic side effects as well as interact with other medications. Hearing therapies and devices are costly and are not efficacious in treating tinnitus without coexisting hearing loss. Medications are systemically administered and lead to unwanted side effects with variable efficacy [67]. 

### Probiotic Effect on Neurotransmitters and Inflammatory Mediators

**Figure 1:**
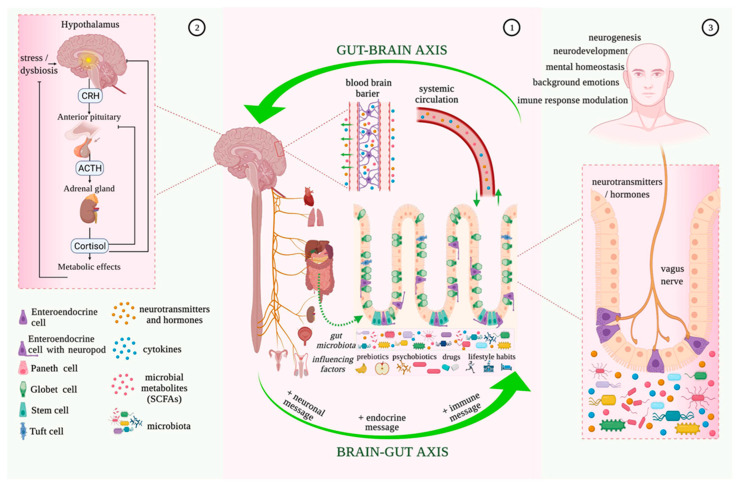
Microbiota–gut–brain bidirectional relationship. Taken from Mitrea et al. (2022) [70] under the Creative Commons Attribution License (CC BY). The use, distribution or reproduction in other forums is permitted, provided the original author(s) and the copyright owner(s) are credited and that the originalpublication in this journal is cited.

Recent studies have shown that the gut microbiome plays a role in regulating the concentration of neurotransmitters like GABA and serotonin, as well as inflammatory mediators like TNF-alpha and IL-6 (Figure 1), potentially contributing to tinnitus [66]. Subsequently, gut dysbiosis leading to decreased GABA has been found to play a role in tinnitus [66]. Thus, one area of further study in the treatment of tinnitus could be to modulate the microbiome to increase GABA and dopamine and decrease TNF-alpha. Luck and colleagues demonstrated that *Bifidobacterium dentium*, which is a member of the healthy gut microbiota that can be isolated from healthy mouse stool, can generate GABA from glutamate or succinate in the intestine [37]. A similar study by Duranti and colleagues indicated that *Bifidobacterium adolescentis* can produce GABA from its precursor monosodium glutamate [38]. Developing probiotics that include these *Bifidobacterium* strains, while observing the associated concentrations of GABA, could therefore be a future field of study in tinnitus patients. As with other auditory disorders, altered neurotransmitter activity can be caused by gut dysbiosis from a high-sugar and high-fat diet and the subsequent increase or decrease in the operational taxonomic units of certain bacteria in the gut [68], which is yet another reason to shift towards healthier diets favoring omega-3 fatty acids. Anti-TNF therapeutics can also be administered, as they have been found to modulate the microbiota by dampening inflammation, inducing T cell apoptosis, and inhibiting vasculitis [69]. Such treatments can also help to reduce the rates of inflammatory bowel disease, which, based on its relationship with the blood–labyrinth barrier, could also protect the integrity of the inner ear and decrease inflammation of the cochlea.

## 8. Conclusions and Future Directions

This review discussedthe modulation of the gut microbiome to improve outcomes in auditory disorders including SNHL, AOM, SOM, and tinnitus. The most effective ways of modulating the gut microbiome to treat auditory disorders included diet, probiotic use, and natural health products. In SNHL, probiotic conversion of nondigestible carbohydrates into short-chain fatty acids could reduce hearing loss by maintaining an adequate vascular supply to the cochlea as well as attenuating inflammatory processes, decreasing blood pressure, and improving vascular reactivity and endothelial function [39,40]. Omega-3 fatty acids, polyphenols, and micronutrients have demonstrated some of the best health benefits in their modulation of the gut microbiota [42,43,44]. Furthermore, combination therapy consisting of probiotic administration alongside a decreased antibiotic dosage has demonstrated positive results in children [42]. In both AOM and SOM, the incorporation of probiotics such as *Lactobacillus* and *Streptococcus* strains to maintain homeostasis in the gut microbiome will help in decreasing antibiotic usage, thus reducing adverse events [50]. Much like in SNHL, studies have suggested that providing a combination therapy of probiotics with a decreased dose of antibiotics yields stronger outcomes than aggressive antibiotic treatment alone [54]. Health products such as combinations of echinacea with propolis and vitamin C, as well as xylitol, have also been able to confer benefits by preventing otitis media [58,59,62]. In treating tinnitus, strains of *Bifidobacterium* have been found to generate GABA from their precursors, and increased GABA can be used to counter the drop in the neurotransmitter seen in gut dysbiosis [64]. 

The modulation of the gut microbiome to develop therapeutic strategies for auditory disorders is still in its infancy stage, and future efficacy studies are warranted. While monounsaturated and omega-3 fatty acids have been found to lower the risk of SNHL in certain populations, the phenomenon has yet to be studied in-depth in elderly populations, which are most susceptible to hearing loss [40]. Many of the studies demonstrated the benefits of combination doses of probiotics and antibiotics in preventing gut dysbiosis that leads to auditory disorders and antibiotic adverse events; however, there was little elaboration on the exact dosages of these combinations. Specific doses of probiotics and antibiotics should be tested in future studies to investigate which combinations present as the most efficacious against each disorder. Future studies should also evaluate whether antibiotics and probiotics decrease the efficacy of each other if they are administered close to one other. This review also introduced the question of oral probiotics in comparison to those administered intranasally; therefore, another direction could be to study how modulating the microbiome of the nasopharynx impacts auditory disorders in comparison to alterations of the gut microbiome. Prebiotics are in the preclinical stage of research and further studies are warranted to determine their efficacy in humans, specifically for audiological disorders. Evaluation of the interplay between probiotics and prebiotics can help clinicians understand their utility as a combination therapy. Lastly, probiotics can be studied and developed to utilize strains of *Bifidobacterium* for increasing levels of GABA in the gut microbiome and help treat tinnitus by maintaining gut homeostasis. Targeting gut dysbiosis holds great potential for the development of effective interventions for auditory disorders by altering neurotransmitter levels and attenuating inflammation that damages sensory structures in the cochlear compartment.

## Figures and Tables

**Table 1:** Gut microbiome modulatory mechanisms and their effects on auditory disorders.

Auditory Disorders	Treatment	Outcome	Authors
Sensorineural Hearing Loss (SNHL)	Polyunsaturated fatty acid consumption	Maintained adequate vascular supply to the cochlea and promotes auditory function	Gopinath et al., 2010 [21]; Fiorini et al., 2016 [22]
	Two or more weekly servings of fish and increased intake of long-chain omega-3 fatty acids	Lower risk of hearing loss in women	Curhan et al., 2014 [23]
	Implementation of the Mediterranean diet	Increased the diversity and stability of the gut microbiome, along with maintaining activity of host immune functions and preventing inflammatory bowel disease	Garcia-Mantrana et al., 2018 [24]; De Filippis et al., 2016 [25]; Rinninela et al., 2019 [26]
Acute Otitis Media (AOM)	Lactobacillus and *Streptococcus*-containing probiotics administered to children	In children not prone to acquiring AOM, the incidence decreased by two-thirds. Also decreased the number of children taking antibiotics for other infections	Scott et al., 2019 [27]
	*Streptococcus salivarius* K12 probiotics administered to children	Decreased incidence of AOM, as well as improved results in tone audiometry, tympanometry, endonasal endoscopy, otoscopy, and tonsillar examination	Di Pierro et al., 2015 [28]
	*Lactobacillus rhamnosus* probiotic and fluoride-supplemented milk administered to children	Otitis media infection duration was reduced by 60%, according to decrease in antibiotic therapy, and further episodes of otitis media (OM) were prevented	Bourdillon et al., 2020 [19]; Stecksen-Blicks et al., 2009 [29]
	*Lactobacillus rhamnosus* GG and *Bifidobacterium lactis* Bb-12 probiotics administered to infants between 2 and 12 months	Largest reduction in OM episodes found within the first 7 months of consuming the supplement. Antibiotic usage reduced from 60% to 31%	Rautava et al., 2008 [30]
	*Streptococcus* probiotic administration via nasal spray	42% of patients (adults and children) did not show recurrence of AOM; children required less antibiotic administration	Roos et al., 2001 [31]; Marchisio et al., 2015 [32]
	Echinacea combined with propolis and vitamin C	Reduced number of AOM episodes by 68%	Cohen et al., 2004 [33]; Wahl et al., 2008 [34]
	Xylitol administered in a gum, syrup, and lozenge	40% decrease in AOM with gum, 30% with syrup, 20% with lozenge; also reduced risk of AOM in children without upper respiratory tract infection	Uhari et al., 1996 [35]; Azarpazhooh et al., 2016 [36]
Secretory Otitis Media (SOM)	*Streptococcus salivarius* K12 administered orally and nasally	Positive treatment results; oral administration conferred improvements in tone audiometry and palatine tonsil size	Bourdillon and Edwards, 2021 [19]
	*Streptococcus sanguinis* administration in children	Increased spontaneous recovery and protection	Czibulka, 2022 [16]
	*Streptococcus sanguinis* administration via nasal spray	Decreased incidence	Czibulka, 2022 [16]
Tinnitus	*Bifidobacterium dentium*	Altered neurotransmitter levels such as gamma-aminobutyric acid (GABA) and serotonin (5-HT); reduced neuroinflammation preventing increased neuronal activity involved in manifestation of tinnitus symptoms	Luck et al., 2021 [37]
	*Bifidobacterium adolescentis*		Duranti et al., 2020 [38]

## Data Availability

Not applicable.

## Abbreviations

5-HT	serotonin
AE	adverse effects
AOM	acute otitis media
FOS	fructo-oligosaccharides
GABA	gamma-aminobutyric acid
GI	gastrointestinal
GOS	galacto-oligosaccharides
IL	interleukin
IT	intratympanic
OM	otitis media
RCT	randomized control trial
SNHL	sensorineural hearing loss
SOM	suppurative otitis media
TNF	tumor necrosis factor
WHO	World health organization

## References

[1] Brody H. (2020). The gut microbiome. Nature.

[2] Fujisaka S., Yoshiyuki W., Tobe K. (2023). The gut microbiome: A core regulator of metabolism. J. Endocrinol..

[3] Heintz-Buschart A., Wilmes P. (2018). Human Gut Microbiome: Function Matters. Trends Microbiol..

[4] Wiefels M.D., Furar E., Eshraghi R.S., Mittal J., Memis I., Moosa M., Mittal R., Eshraghi A. (2022). Targeting Gut Dysbiosis and Microbiome Metabolites for the Development of Therapeutic Modalities for Neurological Disorders. Curr. Neuropharmacol..

[5] Singh R.K., Chang H., Yan D., Lee K.M., Ucmak D., Wong K., Abrouk M., Farahnik B., Nakamura M., Zhu T.H. (2017). Influence of diet on the gut microbiome and implications for human health. J. Transl. Med..

[6] Zysset-Burri D.C., Morandi S., Herzog E.L., Berger L.E., Zinkernagel M.S. (2023). The role of the gut microbiome in eye diseases. Prog. Retin. Eye Res..

[7] Guo C., Che X., Briese T., Ranjan A., Allicock O., Yates R.A., Cheng A., March D., Hornig M., Komaroff A.L. (2023). Deficient butyrate-producing capacity in the gut microbiome is associated with bacterial network disturbances and fatigue symptoms in ME/CFS. Cell Host Microbe.

[8] Xiong R., Gunter C., Fleming E., Vernon S.D., Bateman L., Unutmaz D., Oh J. (2023). Multi-’omics of gut microbiome-host interactions in short- and long-term myalgic encephalomyelitis/chronic fatigue syndrome patients. Cell Host Microbe.

[9] Miyauchi E., Shimokawa C., Steimle A., Desai M.S., Ohno H. (2023). The impact of the gut microbiome on extra-intestinal autoimmune diseases. Nat. Rev. Immunol..

[10] Rampanelli E., Nieuwdorp M. (2023). Gut microbiome in type 1 diabetes: The immunological perspective. Expert. Rev. Clin. Immunol..

[11] Chandra S., Sisodia S.S., Vassar R.J. (2023). The gut microbiome in Alzheimer’s disease: What we know and what remains to be explored. Mol. Neurodegener..

[12] Davies C., Mishra D., Eshraghi R.S., Mittal J., Sinha R., Bulut E., Mittal R., Eshraghi A.A. (2021). Altering the gut microbiome to potentially modulate behavioral manifestations in autism spectrum disorders: A systematic review. Neurosci. Biobehav. Rev..

[13] Eshraghi R.S., Davies C., Iyengar R., Perez L., Mittal R., Eshraghi A.A. (2020). Gut-Induced Inflammation during Development May Compromise the Blood-Brain Barrier and Predispose to Autism Spectrum Disorder. J. Clin. Med..

[14] Eshraghi R.S., Deth R.C., Mittal R., Aranke M., Kay S.S., Moshiree B., Eshraghi A.A. (2018). Early Disruption of the Microbiome Leading to Decreased Antioxidant Capacity and Epigenetic Changes: Implications for the Rise in Autism. Front. Cell Neurosci..

[15] Mittal R., Debs L.H., Patel A.P., Nguyen D., Patel K., O’Connor G., Grati M., Mittal J., Yan D., Eshraghi A.A. (2017). Neurotransmitters: The Critical Modulators Regulating Gut-Brain Axis. J. Cell Physiol..

[16] Czibulka A. (2022). Probiotics for Otolaryngologic Disorders. Otolaryngol. Clin. N. Am..

[17] Denton A.J., Godur D.A., Mittal J., Bencie N.B., Mittal R., Eshraghi A.A. (2022). Recent Advancements in Understanding the Gut Microbiome and the Inner Ear Axis. Otolaryngol. Clin. N. Am..

[18] Rutsch A., Kantsjö J.B., Ronchi F. (2020). The Gut-Brain Axis: How Microbiota and Host Inflammasome Influence Brain Physiology and Pathology. Front. Immunol..

[19] Bourdillon A.T., Edwards H.A. (2021). Review of probiotic use in otolaryngology. Am. J. Otolaryngol..

[20] Haro C., García-Carpintero S., Rangel-Zúñiga O.A., Alcalá-Díaz J.F., Landa B.B., Clemente J.C., Pérez-Martínez P., López-Miranda J., Pérez-Jiménez F., Camargo A. (2017). Consumption of Two Healthy Dietary Patterns Restored Microbiota Dysbiosis in Obese Patients with Metabolic Dysfunction. Mol. Nutr. Food Res..

[21] Gopinath B., Flood V.M., Rochtchina E., McMahon C.M., Mitchell P. (2010). Consumption of omega-3 fatty acids and fish and risk of age-related hearing loss. Am. J. Clin. Nutr..

[22] Fiorini A.C., Costa Filho O.A., Scorza F.A. (2016). Can you hear me now? The quest for better guidance on omega-3 fatty acid consumption to combat hearing loss. Clinics.

[23] Curhan S.G., Eavey R.D., Wang M., Rimm E.R., Curhan G.C. (2014). Fish and fatty acid consumption and the risk of hearing loss in women. Am. J. Clin. Nutr..

[24] Garcia-Mantrana I., Selma-Royo M., Alcantara C., Collado M.C. (2018). Shifts on Gut Microbiota Associated to Mediterranean Diet Adherence and Specific Dietary Intakes on General Adult Population. Front. Microbiol..

[25] De Filippis F., Pellegrini N., Vannini L., Jeffrey I.B., La Storia A., Laghi L., Serrazanetti D.I., Di Cagno R., Ferrocino I., Lazzi C. (2016). High-level adherence to a Mediterranean diet beneficially impacts the gut microbiota and associated metabolome. Gut.

[26] Rinninella E., Cintoni M., Raoul P., Lopetuso L.R., Scaldaferri F., Pulcini G., Miggiano G.A.D., Gasbarrini A., Mele M.C. (2019). Food Components and Dietary Habits: Keys for a Healthy Gut Microbiota Composition. Nutrients.

[27] Scott A.M., Clark J., Julien B., Islam F., Roos K., Grimwood K., Little P., Del Mar C.B. (2019). Probiotics for preventing acute otitis media in children. Cochrane Database Syst. Rev..

[28] Di Pierro F., Di Pasquale D., Di Cicco M. (2015). Oral use of Streptococcus salivarius K12 in children with secretory otitis media: Preliminary results of a pilot, uncontrolled study. Int. J. Gen. Med..

[29] Stecksén-Blicks C., Sjöström I., Twetman S. (2009). Effect of long-term consumption of milk supplemented with probiotic lactobacilli and fluoride on dental caries and general health in preschool children: A cluster-randomized study. Caries Res..

[30] Rautava S., Salminen S., Isolauri E. (2009). Specific probiotics in reducing the risk of acute infections in infancy--a randomized, double-blind, placebo-controlled study. Br. J. Nutr..

[31] Roos K., Håkansson E.G., Holm S. (2001). Effect of recolonisation with interfering alpha streptococci on recurrences of acute and secretory otitis media in children: Randomised placebo controlled trial. BMJ.

[32] Marchisio P., Santagati M., Scillato M., Baggi E., Fattizzo M., Rosazza C., Stefani S., Esposito S., Principi N. (2015). Streptococcus salivarius 24SMB administered by nasal spray for the prevention of acute otitis media in otitis-prone children. Eur. J. Clin. Microbiol. Infect. Dis..

[33] Cohen H.A., Varsano I., Kahan E., Sarrell E.M., Uziel Y. (2004). Effectiveness of an herbal preparation containing echinacea, propolis, and vitamin C in preventing respiratory tract infections in children: A randomized, double-blind, placebo-controlled, multicenter study. Arch. Pediatr. Adolesc. Med..

[34] Wahl R.A., Aldous M.B., Worden K.A., Grant K.L. (2008). Echinacea purpurea and osteopathic manipulative treatment in children with recurrent otitis media: A randomized controlled trial. BMC Complement. Altern. Med..

[35] Uhari M., Kontiokari T., Koskela M., Niemelä M. (1996). Xylitol chewing gum in prevention of AOM: Double-blind randomised trials. Br. Med. J..

[36] Azarpazhooh A., Lawrence H.P., Shah P.S. (2016). Xylitol for preventing acute otitis media in children up to 12 years of age. Cochrane Database Syst. Rev..

[37] Luck B., Horvath T., Engevik K.A., Ruan W., Haidacher S.J., Hoch K.M., Oezguen N., Spinler J.K., Haag A.M., Versalovic J. (2021). Neurotransmitter Profiles Are Altered in the Gut and Brain of Mice Mono-Associated with Bifidobacterium dentium. Biomolecules.

[38] Duranti S., Ruuiz L., Lugli G.A., Tames H., Milani C., Mancabelli L., Mancino W., Carnevali L., Sgoifo A., Margolles A. (2020). Bifidobacterium adolescentis as a key member of the human gut microbiota in the production of GABA. Sci. Rep..

[39] Mack D.R. (2005). Probiotics-mixed messages. Can. Fam. Physician.

[40] Yadav M.K., Kumari I., Singh B., Sharma K.K., Tiwari S.K. (2022). Probiotics, prebiotics and synbiotics: Safe options for next-generation therapeutics. Appl. Microbiol. Biotechnol..

[41] Kim S.K., Guevarra R.B., Kim Y.T., Kwon J., Kim H., Cho J.H., Kim H.B., Lee J.H. (2019). Role of Probiotics in Human Gut Microbiome-Associated Diseases. J. Microbiol. Biotechnol..

[42] Wieërs G., Belkhir L., Enaud R., Leclercq S., de Foy J.M.P., Dequenne I., de Timary P., Cani P.D. (2019). How Probiotics Affect the Microbiota. Front. Cell Infect. Microbiol..

[43] Suez J., Zmora N., Segal E., Elinav E. (2019). The pros, cons, and many unknowns of probiotics. Nat. Med..

[44] van Zyl W.F., Deane S.M., Dicks L.M.T. (2020). Molecular insights into probiotic mechanisms of action employed against intestinal pathogenic bacteria. Gut Microbes.

[45] Nathan A.S., Levi J.R., O’Reilly R. (2022). Complementary/Integrative Medicine for Pediatric Otitis Media. Otolaryngol. Clin. North. Am..

[46] Davani-Davari D., Negahdaripour M., Karimzadeh I., Seifan M., Mohkam M., Masoumi S.J., Berenjian A., Ghasemi Y. (2019). Prebiotics: Definition, Types, Sources, Mechanisms, and Clinical Applications. Foods.

[47] Xie X., He Y., Li H., Yu D., Na L., Sun T., Zhang D., Shi X., Xia Y., Jiang T. (2019). Effects of prebiotics on immunologic indicators and intestinal microbiota structure in perioperative colorectal cancer patients. Nutrition.

[48] Hall D.A., Voigt R.M., Cantu-Jungles T.M., Hamaker B., Engen P.A., Shaikh M., Raeisi S., Green S.J., Naqib A., Forsyth C.B. (2023). An open label, non-randomized study assessing a prebiotic fiber intervention in a small cohort of Parkinson’s disease participants. Nat. Commun..

[49] Birkeland E., Gharagozlian S., Birkeland K.I., Valeur J., Måge I., Rud I., Aas A.-M. (2020). Prebiotic effect of inulin-type fructans on faecal microbiota and short-chain fatty acids in type 2 diabetes: A randomised controlled trial. Eur. J. Nutr..

[50] Buhaș M.C., Candrea R., Gavrilaș L.I., Miere D., Tătaru A., Boca A., Cătinean A. (2023). Transforming Psoriasis Care: Probiotics and Prebiotics as Novel Therapeutic Approaches. Int. J. Mol. Sci..

[51] Slavin J. (2013). Fiber and prebiotics: Mechanisms and health benefits. Nutrients.

[52] Kondo T., Saigo S., Ugawa S., Kato M., Yoshikawa Y., Miyoshi N., Tanabe K. (2020). Prebiotic effect of fructo-oligosaccharides on the inner ear of DBA/2 J mice with early-onset progressive hearing loss. J. Nutr. Biochem..

[53] Cervin A.U. (2017). The Potential for Topical Probiotic Treatment of Chronic Rhinosinusitis, a Personal Perspective. Front. Cell Infect. Microbiol..

[54] Cai Y., Juszczak H.M., Cope E.K., Goldberg A.N. (2021). The microbiome in obstructive sleep apnea. Sleep.

[55] Kociszewska D., Vlajkovic S.M. (2022). The Association of Inflammatory Gut Diseases with Neuroinflammatory and Auditory Disorders. Front. Biosci..

[56] Sujlana A., Goyal R., Pannu P., Opal S., Bansal P. (2017). Visual pedagogy and probiotics for hearing impaired children: A pilot study. J. Indian. Soc. Pedod. Prev. Dent..

[57] Kaliannan K., Wang B., Li X.Y., Kim K.J., Kang J.X. (2015). A host-microbiome interaction mediates the opposing effects of omega-6 and omega-3 fatty acids on metabolic endotoxemia. Sci. Rep..

[58] Menni C., Zierer J., Pallister T., Jackson M.A., Long T., Mohney R.P., Steves C.J., Spector T.D., Valdes A.M. (2017). Omega-3 fatty acids correlate with gut microbiome diversity and production of N-carbamylglutamate in middle aged and elderly women. Sci. Rep..

[59] Mittal R., Parrish J.M., Soni M., Mittal J., Mathee K. (2018). Microbial otitis media: Recent advancements in treatment, current challenges and opportunities. J. Med. Microbiol..

[60] Venekamp R.P., Sanders S.L., Glasziou P.P., Del Mar C.B., Rovers M.M. (2015). Antibiotics for acute otitis media in children. Cochrane Database Syst. Rev..

[61] Lukasik J., Patro-Golab B., Horvath A., Baron R., Szajewska H., SAWANTI Working Group (2019). Early Life Exposure to Antibiotics and Autism Spectrum Disorders: A Systematic Review. J. Autism Dev. Disord..

[62] Reid G. (2006). Probiotics to prevent the need for, and augment the use of, antibiotics. Can. J. Infect. Dis. Med. Microbiol..

[63] Karsch-Völk M., Barrett B., Kiefer D., Bauer R., Ardjomand-Woelkart K., Linde K. (2014). Echinacea for preventing and treating the common cold. Cochrane Database Syst. Rev..

[64] Schapowal A., Klein P., Johnston S.L. (2015). Echinacea reduces the risk of recurrent respiratory tract infections and complications: A meta-analysis of randomized controlled trials. Adv. Ther..

[65] Ogal M., Johnston S.L., Klein P., Schoop R. (2021). Echinacea reduces antibiotic usage in children through respiratory tract infection prevention: A randomized, blinded, controlled clinical trial. Eur. J. Med. Res..

[66] Megantara I., Wikargana G.L., Dewi Y.A., Permana A.D., Sylviana N. (2022). The Role of Gut Dysbiosis in the Pathophysiology of Tinnitus: A Literature Review. Int. Tinnitus J..

[67] Baguley D., McFerran D., Hall D. (2013). Tinnitus. Lancet.

[68] Guo Y., Zhu X., Zeng M., Qi L., Tang X., Wang D., Zhang M., Xie Y., Li H., Yang X. (2021). A diet high in sugar and fat influences neurotransmitter metabolism and then affects brain function by altering the gut microbiota. Transl. Psychiatry.

[69] Jones-Hall Y.L., Nakatsu C.H. (2016). The Intersection of TNF, IBD and the Microbiome. Gut Microbes.

[70] Mitrea L., Nemeş S.A., Szabo K., Teleky B.E., Vodnar D.C. (2022). Guts imbalance imbalances the brain: A review of gut microbiota association with neurological and psychiatric disorders. Front. Med..

